# Laparoscopic versus open pancreaticoduodenectomy for pancreatic ductal adenocarcinoma: a propensity score matching analysis

**DOI:** 10.1186/s40880-019-0410-8

**Published:** 2019-10-28

**Authors:** Wentao Zhou, Weiwei Jin, Dansong Wang, Chao Lu, Xuefeng Xu, Renchao Zhang, Tiantao Kuang, Yucheng Zhou, Wenchuan Wu, Dayong Jin, Yiping Mou, Wenhui Lou

**Affiliations:** 1 Department of General Surgery, Zhongshan Hospital, Fudan University, 180 Fenglin Road, Xuhui District, Shanghai, 200032 P. R. China; 2 Department of Gastrointestinal and Pancreatic Surgery, Zhejiang Provincial People’s Hospital, Key Laboratory of Gastroenterology of Zhejiang Province, People’s Hospital of Hangzhou Medical College, 158 Shangtang Road, Hangzhou, 310014 Zhejiang P. R. China

**Keywords:** Laparoscopy, Open pancreaticoduodenectomy, Pancreatic cancer, Overall survival, Gastric emptying, Complications, Adjuvant chemotherapy, Propensity score matching

## Abstract

**Background:**

A growing body of evidence supports the use of laparoscopic pancreaticoduodenectomy (LPD) as an efficient and feasible surgical technique. However, few studies have investigated its applicability in pancreatic ductal adenocarcinoma (PDAC), and the long-term efficacy of LPD on PDAC remains unclear. This study aimed to compare the short- and long-term outcomes between LPD and open pancreaticoduodenectomy (OPD) for PDAC.

**Methods:**

The data of patients who had OPD or LPD for PDAC between January 2013 and September 2017 were retrieved. Their postoperative outcomes and survival were compared after propensity score matching.

**Results:**

A total of 309 patients were included. After a 2:1 matching, 93 cases in the OPD group and 55 in the LPD group were identified. Delayed gastric emptying (DGE), particularly grade B/C DGE, occurred less frequently in the LPD group than in the OPD group (1.8% vs. 36.6%, *P* < 0.001; 1.8% vs. 22.6%, *P* = 0.001). The overall complication rates were significantly lower in the LPD group than in the OPD group (49.1% vs. 71.0%, *P* = 0.008), whereas the rates of major complications were similar (10.9% vs. 14.0%, *P* = 0.590). In addition, the median overall survival was comparable between the two groups (20.0 vs. 18.7 months, *P* = 0.293).

**Conclusion:**

LPD was found to be technically feasible with efficacy similar to OPD for patients with PDAC.

## Background

Pancreatic ductal adenocarcinoma (PDAC) is a common malignancy and is estimated to become the second leading cause of cancer-related deaths by 2030 [1, 2]. At present, surgical resection represents a potentially curative therapy for PDAC, especially for resectable cases. In recent years, advancements in minimally invasive techniques have revolutionized surgeries for pancreatic cancers [3, 4]. Several studies have demonstrated that laparoscopic procedures are both feasible and safe when applied to distal pancreatectomy and can result in similar oncological outcomes as compared with open surgery for PDAC [5–8]. Therefore, laparoscopic surgery has become a routine alternative for malignancies located in the body and tail of the pancreas in many institutions. However, the laparoscopic approach has been slow to gain popularity for patients with pancreatic head cancers even though the feasibility of this technique was first validated in 1994 by Gagner and Pomp [9]. This may, at least in part, be due to the procedural complexity of this technique, as well as oncological uncertainty.

Over recent years, a growing body of literature relating to the comparison of perioperative outcomes between laparoscopic pancreaticoduodenectomy (LPD) and open pancreaticoduodenectomy (OPD) have been published [10–14]. Nevertheless, the vast majority of such reports consisted of small sample sizes, included cases with multiple pathological diagnoses, and were carried out in low-volume institutions, and thus the reported data may not be representative of the real-world clinical situation. In addition, no study compared the long-term efficacy of the two procedures on PDAC. Both Croome et al. [15] and Stauffer et al. [16] reported that LPD was comparable with OPD for PDAC in terms of short-time outcomes and long-term survival. However, neither of these studies considered the potential consequences of confounding factors or selection bias. We therefore performed a propensity score matching study aiming to compare the postoperative outcomes and survival of PDAC patients who underwent LPD or OPD, with well-balanced confounding factors.

## Patients and methods

### Patient selection

First, we reviewed the medical records of consecutive patients who underwent OPD at Zhongshan Hospital, Fudan University (Shanghai, China) or LPD at Zhejiang Provincial People’s Hospital (Hangzhou, Zhejiang, China) between January 2013 and September 2017. Patients with pathologically confirmed PDAC and without any evidence of distant metastasis by preoperative examinations were included. All of the included cases met the resectable criteria laid down by the National Comprehensive Cancer Network guidelines for preoperative assessments [17]. We (YP Mou, RC Zhang and YC Zhou) began to perform LPD in 2012, and more than 10 LPDs were completed for less aggressive pancreatic tumors, such as neuroendocrine tumors and cystic neoplasms, in that year. OPDs were performed by five surgeons (DY Jin, WH Lou, DS Wang, WC Wu and TT Kuang). This research was approved by the Ethics Committee of both Zhongshan Hospital and Zhejiang Provincial People’s Hospital. Both hospitals are high-volume pancreatic surgical centers, and the surgical teams are both experienced in open and laparoscopic surgery.

### Variables and definitions

Demographic, clinical, and pathological data were extracted from corresponding medical records. Baseline characteristics included patient age, gender, body mass index, American Society of Anesthesiologists score, Charlson comorbidity index [18], year of operation, tumor differentiation, nerve invasion, T stage, N stage, TNM stage, and history of adjuvant treatment. TNM stage was classified according to the American Joint Committee on Cancer staging system (8th edition) [19]. Adjuvant treatment comprised of postoperative chemotherapy (e.g., gemcitabine, S−1) or chemoradiotherapy (e.g., gemcitabine plus radiotherapy). The primary endpoint was median overall survival (OS). OS was defined as the duration from the first day after surgery to either the date of death or the last follow-up. Secondary endpoints included postoperative complications, digital subtraction angiography (DSA) intervention, reoperation, in-hospital mortality, readmission, postoperative length of stay, and time to adjuvant chemotherapy. Complications were evaluated based on the Clavien-Dindo classification system [20], and the highest grade for each patient was analyzed for overall postoperative complications. Postoperative pancreatic fistula (POPF) [21], delayed gastric emptying (DGE) [22], and postpancreatectomy hemorrhage (PPH) [23] were defined and classified according to the criteria set out by the International Study Group of Pancreatic Surgery (ISGPS). Similarly, bile leakage (BL) was recorded and graded according to the standard definitions of the International Study Group of Liver Surgery [24]. Wound infection was defined as purulent drainage from the incision or/and positive findings of culture of the fluid or tissue aseptically obtained from the incision. Operative details, such as duration of the operation, estimated blood loss, intraoperative blood transfusion, vascular resection, number of resected lymph nodes, and R0 resection rate, were also analyzed. R0 resection was defined as the absence of tumor cells on the pancreatic neck margin, the retroperitoneal margin, and the bile duct margin. The definitions for all these parameters were unified by both teams at the beginning of this study.

### Surgical technique

The technique we used for LPD was as described in a previous publication [25]. Briefly, five trocars were placed in the abdomen in a V shape. If there was no sign of metastasis upon laparotomy, the gastrocolic ligament was divided to enter the lesser omental sac and expose the anterior surface of the pancreas. Then, the right gastroepiploic vessels were isolated and excised. After dissecting along the superior border of the pancreas, the common hepatic artery, gastroduodenal artery, and proper hepatic artery were identified, and the gastroduodenal artery was further ligated. The inferior border of the pancreas was then dissected to expose the portal vein and superior mesenteric vein, and a retropancreatic tunnel was established prior to the Kocher maneuver. The proximal jejunum and distal stomach or proximal duodenum were divided with liner staplers, then the gallbladder was isolated, and the common hepatic duct was transected with scissors. Subsequently, the pancreatic neck and uncinate process were divided using ultrasonic shears. Finally, the specimen was placed into an endoscopic bag for retrieval. For reconstruction, the Child’s procedure was used, involving pancreaticojejunostomy, hepaticojejunostomy, and gastrojejunostomy in a sequential order. An internal stent was then used to maneuver an end-to-side, duct-to-mucosa pancreaticojejunostomy. This was followed by an end-to-side hepaticojejunostomy using a 4-0 absorbable suture. Subsequently, an end-to-side gastrojejunostomy was performed in an antecolic type using a stapler.

Our OPD procedure resembled the LPD procedure except for two aspects. First, we mainly performed the Kocher maneuver as an initial step after negative abdominal exploration. Second, various fashions of pancreaticojejunostomy were adopted by our OPD team depending on the surgeon’s individual preferences.

### Postoperative treatment

For LPD patients, we routinely stopped using antibiotics at 2 days after surgery if there were no definite POPF, BL, or infections. The nasogastric tube was usually removed on the first or second postoperative day if the volume of digestive juice was less than 200 mL/day and had a normal appearance. The patients were then encouraged to take a liquid diet, followed by a semi-liquid diet. Amylase measurements of the drainage fluid were conducted since the first postoperative day, and the drainage tubes were removed if the volume was less than 50 mL/day for three consecutive days and the amylase level was lower than three times the upper normal serum amylase level and had a normal appearance.

The postoperative treatments in the OPD group shared similarities with those in the LPD group. However, our OPD team usually removed the nasogastric tubes on the third or fourth postoperative day in consideration of the relatively late recovery of gastrointestinal motility following open surgery. In addition, abdominal computed tomography (CT) scan was routinely performed before the removal of drainage tubes for patients in the OPD group, but not routinely performed for patients in the LPD group.

### Follow-up

Patients were recommended to return to the outpatient department for follow-up 1 month after being discharged, every 3–6 months for the first 2 years, then annually. We routinely performed a variety of tests, including blood tests, liver and kidney function tests, carbohydrate antigen 19-9 detection, and chest X-rays; abdominal CT scans were performed when appropriate. Survival data were collected by searching the electronical outpatient system or by telephone interviews. The last follow-up was in November 2017.

### Statistical analyses

To minimize the effect of confounding factors and potential bias between the OPD and LPD groups, propensity score was calculated using logistic regression, and a 2:1 patient matching was performed using the nearest-neighbor matching method without replacement. A caliper radius equal to a standard deviation of 0.1 was set to prevent poor matching. Variables included in the matching model were gender, tumor differentiation, nerve invasion, T stage, and adjuvant treatment; these were distributed differently between the two original groups.

Continuous variables are described as medians and interquartile ranges (IQR), while categorical variables are expressed as whole numbers and percentages. Two-tailed unpaired *t* tests were performed to compare the continuous variables that followed normal distributions; otherwise, the Mann–Whitney *U* test was used. The distribution differences of categorical variables between the two groups were analyzed using the Pearson Chi square tests or Fisher’s exact tests. Survival analyses were conducted using the Kaplan–Meier method with log-rank tests. Univariable and multivariable Cox regression analyses were used to identify independent risk factors of OS. All statistical analyses were performed with SPSS software (version 22.0, IBM Corp., Armonk, NY, USA). Propensity score matching (PSM) was carried out using the “PS MATCHING 3.04”, “SPSS Statistics R Essentials 22.0”, and “R-2.15.3-win” R packages. The GraphPad Prism software (version 5.01, GraphPad Software Inc., San Diego, CA, USA) was used to plot the OS curves. All *P* values were based on 2-sided statistical analyses, and *P* < 0.05 was considered significant.

## Results

### Patient selection and matching

**Fig. 1:**
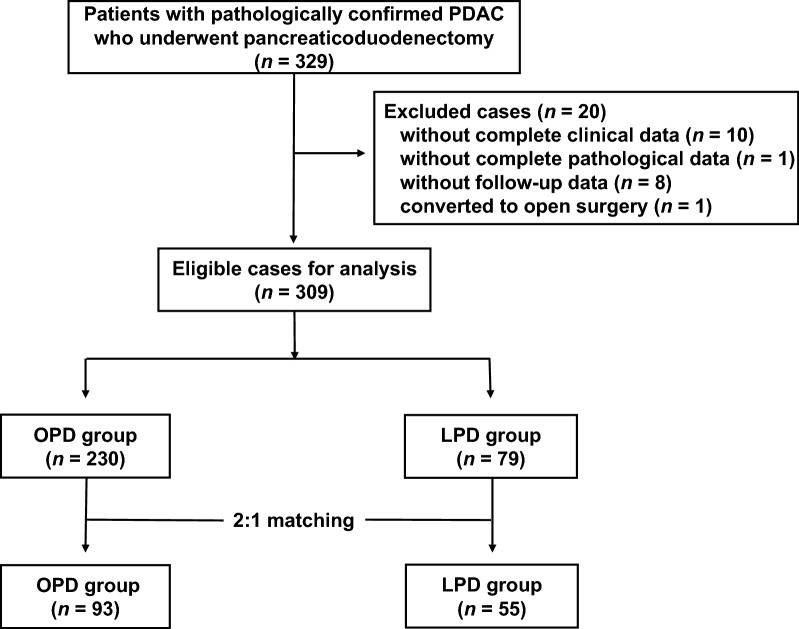
Flow chart of the patient selection process. *PDAC* pancreatic ductal adenocarcinoma, *OPD* open pancreaticoduodenectomy, *LPD* laparoscopic pancreaticoduodenectomy

A total of 329 patients met the inclusion criteria and were included in this study. After excluding cases due to missing data (*n* = 19) and conversion from LPD to OPD (*n* = 1), 309 patients were included for analyses (Fig. 1). The entire cohort consisted of 110 females and 199 males with a median age of 64 years (IQR, 57–70 years). No patients underwent neoadjuvant chemotherapy. Significant differences were observed in baseline characteristics between the OPD group (*n* = 230) and the LPD group (*n* = 79) in the original cohort. After PSM was performed, 93 patients in the OPD group and 55 in the LPD group composed the matched cohort (Table 1).

**Table 1:** Comparison of baseline characteristics between PDAC patients in the OPD and LPD groups

Characteristic	Original cohort		*P*	Matched cohort		*P*
	OPD group	LPD group		OPD group	LPD group	
Total (cases)	230	79		93	55	
Age [years, median (IQR)]	64.0 (57.0–69.3)	64.0 (56.0–70.0)	0.817	64.0 (59.0–70.5)	63.0 (54.0–69.0)	0.337
Gender [cases (%)]			0.027			0.959
Male	140 (60.9)	59 (74.7)		68 (73.1)	40 (72.7)	
Female	90 (39.1)	20 (25.3)		25 (26.9)	15 (27.3)	
BMI [kg/m^2^, median (IQR)]	22.5 (20.5–24.2)	23.2 (20.7–25.3)	0.100	22.3 (20.3–23.9)	23.0 (20.7–25.2)	0.107
ASA score [cases (%)]			0.059			0.149
1	43 (18.7)	6 (7.6)		18 (19.4)	5 (9.1)	
2	177 (77.0)	68 (86.1)		73 (78.5)	47 (85.5)	
3	10 (4.3)	5 (6.3)		2 (2.2)	3 (5.5)	
Charlson comorbidity index [cases (%)]			0.324			0.356
0	144 (62.6)	44 (55.7)		58 (62.4)	31 (56.4)	
1	64 (27.8)	29 (36.7)		28 (30.1)	22 (40.0)	
≥ 2	22 (9.6)	6 (7.6)		7 (7.5)	2 (3.6)	
Year of operation [cases (%)]			0.256			0.447
2013–2015	114 (49.6)	45 (57.0)		55 (59.1)	29 (52.7)	
2016–2017	116 (50.4)	34 (43.0)		38 (40.9)	26 (47.3)	
Tumor differentiation [cases (%)]			0.006			0.421
Well-moderate	82 (35.7)	42 (53.2)		41 (44.1)	28 (50.9)	
Poor	148 (64.3)	37 (46.8)		52 (55.9)	27 (49.1)	
Nerve invasion [cases (%)]			< 0.001			0.131
Yes	203 (88.3)	44 (55.7)		73 (78.5)	37 (67.3)	
No	27 (11.7)	35 (44.3)		20 (21.5)	18 (32.7)	
T stage [cases (%)]			0.001			0.835
1	61 (26.5)	12 (15.2)		15 (16.1)	10 (18.2)	
2	158 (68.7)	54 (68.4)		73 (78.5)	41 (74.5)	
3	11 (4.8)	13 (16.5)		5 (5.4)	4 (7.3)	
N stage [cases (%)]			0.942			0.886
0	123 (53.5)	44 (55.7)		50 (53.8)	32 (58.2)	
1	86 (37.4)	28 (35.4)		35 (37.6)	19 (34.5)	
2	21 (9.1)	7 (8.9)		8 (8.6)	4 (7.3)	
AJCC TNM stage [cases (%)]			0.794			0.831
I	117 (50.9)	37 (46.8)		46 (49.5)	30 (54.5)	
II	92 (40.0)	35 (44.3)		39 (41.9)	21 (38.2)	
III	21 (9.1)	7 (8.9)		8 (8.6)	4 (7.3)	
Adjuvant treatment [cases (%)]			< 0.001			0.701
Yes	162 (70.4)	31 (39.2)		47 (50.5)	26 (47.3)	
No	68 (29.6)	48 (60.8)		46 (49.5)	29 (52.7)	

*PDAC* pancreatic ductal adenocarcinoma, *OPD* open pancreaticoduodenectomy, *LPD* laparoscopic pancreaticoduodenectomy, *IQR* interquartile range, *BMI* body mass index, *ASA* American Society of Anesthesiologists score, *AJCC* American Joint Committee on Cancer. The 8th edition of AJCC TNM staging system was used

### Operative characteristics

Surgical characteristics of patients in the LPD and OPD groups are shown in Table 2. In the original cohort, LPD was associated with significantly longer operative time (330.0 vs. 260.5 min, *P* < 0.001), lower amount of estimated blood loss (150.0 vs. 200.0 mL, *P* < 0.001), higher frequency of intraoperative blood transfusion (24.1% vs. 10.4%, *P* = 0.003), and more resected lymph nodes (18.0 vs. 11.0, *P* < 0.001). No patients underwent vessel resection in this study. In the matched cohort, LPD was still associated with significantly longer operative time (330.0 vs. 260.0 min, *P* < 0.001), lower amount of estimated blood loss (150.0 vs. 200.0 mL, *P* = 0.001), and more resected lymph nodes (18.0 vs. 11.0, *P* < 0.001). However, a significantly higher frequency of blood transfusion was observed in the LPD group (29.1% vs. 7.5%, *P* < 0.001).

**Table 2:** Comparison of operative characteristics of PDAC patients between the OPD and LPD groups

Characteristic	Original cohort		*P*	Matched cohort		*P*
	OPD group (*n* = 230)	LPD group (*n* = 79)		OPD group (*n* = 93)	LPD group (*n* = 55)	
Operative time [min, median (IQR)]	260.5 (210.0–360.0)	330.0 (260.0–376.0)	< 0.001	260.0 (207.5–325.5)	330.0 (260.0–360.0)	< 0.001
Estimated blood loss [mL, median (IQR)]	200.0 (100.0–400.0)	150.0 (100.0–200.0)	< 0.001	200.0 (150.0–350.0)	150.0 (100.0–200.0)	0.001
Blood transfusion [cases (%)]	24 (10.4)	19 (24.1)	0.003	7 (7.5)	16 (29.1)	< 0.001
No. of resected lymph nodes [median (IQR)]	11.0 (7.0–15.0)	18.0 (14.0–22.0)	< 0.001	11.0 (7.0–14.5)	18.0 (13.0–25.0)	< 0.001
No. of positive lymph nodes [median (IQR)]	0 (0–2.0)	0 (0–2.0)	0.761	0 (0–2.0)	0 (0–2.0)	0.909
Positive lymph node ratio [median (IQR)]	0 (0–0.14)	0 (0–0.09)	0.353	0 (0–0.16)	0 (0–0.08)	0.366
R0 resection [cases (%)]	221 (96.1)	79 (100)	0.163	88 (94.6)	55 (100)	0.201

*PDAC* pancreatic ductal adenocarcinoma, *OPD* open pancreaticoduodenectomy, *LPD* laparoscopic pancreaticoduodenectomy, *IQR* interquartile range

### Postoperative outcomes assessment

Detailed comparisons of postoperative outcomes are listed in Table 3. In the original cohort, the rate of postoperative biochemical leak was significantly lower in the LPD group than in the OPD group (16.5% vs. 32.2%, *P* = 0.007), although the rate of POPF was not significantly different between the two groups (25.3% vs. 37.4%, *P* = 0.051). Delayed gastric emptying, both grade A and grade B/C, occurred significantly less commonly in the LPD group than in the OPD group (overall, 1.3% vs. 28.7%, *P* < 0.001; biochemical leak, 0 vs. 11.7%, *P* = 0.001; grade B/C, 1.3% vs. 17.0%, *P* < 0.001). No significant difference was observed in terms of other complications between the two groups. The overall complication rate was significantly lower in the LPD group than in the OPD group (49.4% vs. 65.7%, *P* = 0.010), which was related with the lower rate of minor complications (Clavien I–II) in the LPD group (38.0% vs. 57.8%, *P* = 0.002). However, compared to OPD, LPD was associated with a higher rate of reoperation (1.3% vs. 6.3%, *P* = 0.044).

**Table 3:** Comparison of postoperative outcomes between PDAC patients in the OPD and LPD groups

Characteristic	Original cohort		*P*	Matched cohort		*P*
	OPD group (*n* = 230)	LPD group (*n* = 79)		OPD group (*n* = 93)	LPD group (*n* = 55)	
POPF [cases (%)]	86 (37.4)	20 (25.3)	0.051	33 (35.5)	13 (23.6)	0.132
Biochemical leak	74 (32.2)	13 (16.5)	0.007	28 (30.1)	9 (16.4)	0.062
Grade B/C	12 (5.2)	7 (8.9)	0.373	5 (5.4)	4 (7.3)	0.912
DGE [cases (%)]	66 (28.7)	1 (1.3)	< 0.001	34 (36.6)	1 (1.8)	< 0.001
Grade A	27 (11.7)	0 (0.0)	0.001	13 (14.0)	0 (0.0)	0.009
Grade B/C	39 (17.0)	1 (1.3)	< 0.001	21 (22.6)	1 (1.8)	0.001
PPH [cases (%)]	14 (6.1)	6 (7.6)	0.638	10 (10.8)	4 (7.3)	0.485
Grade A	5 (2.2)	1 (1.3)	0.974	4 (4.3)	0 (0.0)	0.301
Grade B/C	9 (3.9)	5 (6.3)	0.564	6 (6.5)	4 (7.3)	1.000
BL [cases (%)]	15 (6.5)	6 (7.6)	0.744	5 (5.4)	6 (10.9)	0.360
Grade A	5 (2.2)	0 (0.0)	0.421	2 (2.2)	0 (0.0)	0.530^a^
Grade B/C	10 (4.3)	6 (7.6)	0.407	3 (3.2)	6 (10.9)	0.125
Intra-abdominal infection [cases (%)]	21 (9.1)	4 (5.1)	0.253	12 (12.9)	2 (3.6)	0.063
Wound infection [cases (%)]	3 (1.3)	4 (5.1)	0.134	0 (0.0)	2 (3.6)	0.137^a^
Other complications [cases (%)]	24 (10.4)	8 (10.1)	0.938	11 (11.8)	6 (10.9)	0.865
Overall complications [cases (%)]	151 (65.7)	39 (49.4)	0.010	66 (71.0)	27 (49.1)	0.008
Clavien I–II	133 (57.8)	30 (38.0)	0.002	53 (57.0)	21 (38.2)	0.027
Clavien III–V	18 (7.8)	9 (11.4)	0.333	13 (14.0)	6 (10.9)	0.590
DSA [cases (%)]	7 (3.0)	5 (6.3)	0.334	5 (5.4)	4 (7.3)	0.912
Reoperation [cases (%)]	3 (1.3)	5 (6.3)	0.044	1 (1.1)	2 (3.6)	0.642
In-hospital mortality [cases (%)]	2 (0.9)	1 (1.3)	1.000^a^	2 (2.2)	0 (0.0)	0.530^a^
Readmission [cases (%)]	32 (13.9)	8 (10.1)	0.387	14 (15.1)	6 (10.9)	0.476
Postoperative length of stay [days, median (IQR)]	13.0 (10.0–19.0)	12.0 (10.0–18.0)	0.947	14.0 (10.0–20.0)	13.0 (11.0–20.0)	0.986
Time to adjuvant chemotherapy [days, median (IQR)]	44.0 (33.8–63.0)	39.0 (32.0–77.0)	0.616	43.5 (32.8–57.3)	39.0 (32.5–81.0)	0.935

*PDAC* pancreatic ductal adenocarcinoma, *OPD* open pancreaticoduodenectomy, *LPD* laparoscopic pancreaticoduodenectomy, *POPF* postoperative pancreatic fistula, *DGE* delayed gastric emptying, *PPH* post-pancreatectomy hemorrhage, *BL* bile leakage, *DSA* digital subtraction angiography, *IQR* interquartile range

^a^Fisher’s exact test

After matching, the rates of both biochemical leak and grade B/C DGE were still significantly lower in the LPD group than in the OPD group (0 vs. 14.0%, *P* = 0.009; 1.8% vs. 22.6%, *P* = 0.001). There were no other significant differences in complications between the two groups. Overall, LPD was associated with significant reduction in minor complications (Clavien I–II, 38.2% vs. 57.0%, *P* = 0.027), but similar rate of major complications (Clavien III–V, 10.9% vs. 14.0%, *P* = 0.590) as to the OPD group.

### Overall survival and prognostic factors

**Fig. 2:**
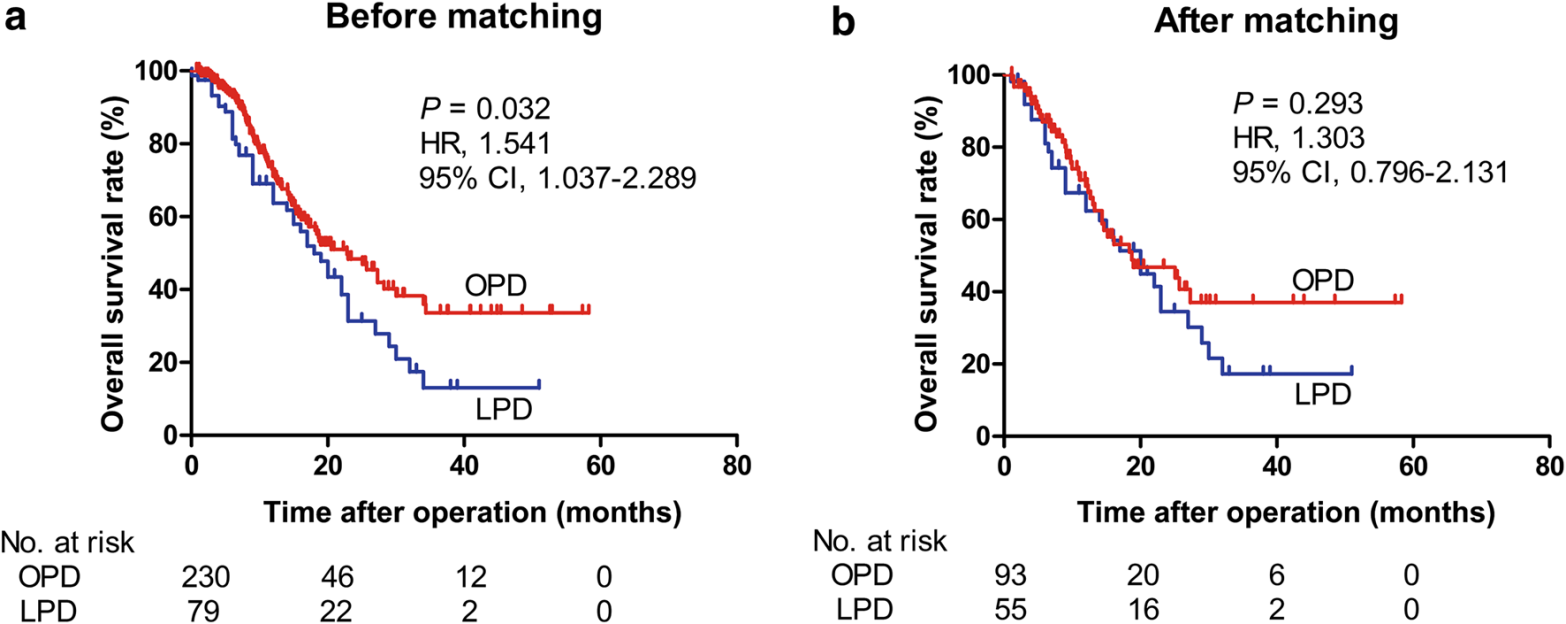
Kaplan–Meier curves for OS comparison of patients undergoing OPD versus LPD for PDAC. **a** Before propensity score matching. **b** After propensity score matching. *OS* overall survival, *OPD* open pancreaticoduodenectomy, *LPD* laparoscopic pancreaticoduodenectomy, *PDAC* pancreatic ductal adenocarcinoma, *HR* hazard ratio, *CI* confidence interval

By November 2017, 130 patients (42.1%) had died, 139 (45.0%) remained alive, and 40 (12.9%) were lost to follow-up. Those who were lost to follow-up were considered to be alive on the day before the last recorded follow-up when their data were handled in survival analysis. The median follow-up period was 11.2 (IQR, 6.4–18.8) months. Before matching, the median OS was 18.0 months in the LPD group and 22.8 months in the OPD group (hazard ratio [HR] = 1.541, 95% confidence interval [CI] = 1.037–2.289, *P* = 0.032) (Fig. 2a). After matching, the median OS was 20.0 and 18.7 months in the LPD and OPD groups, respectively (HR = 1.303, 95% CI = 0.796–2.131, *P* = 0.293) (Fig. 2b). Univariate Cox regression analysis showed that tumor differentiation (*P* = 0.001), T stage (*P* = 0.028), N stage (*P* = 0.042), TNM stage (*P* = 0.025), and adjuvant treatment (*P* < 0.001) were associated with OS in the original cohort. However, only tumor differentiation (HR = 2.020, 95% CI = 1.369–2.984, *P* < 0.001) and adjuvant treatment (HR = 0.364, 95% CI = 0.245–0.539, *P* < 0.001) were validated as independent risk factors by multivariate analysis (Table 4), both of which were well matched in the previous comparison analysis of the median OS.

**Table 4:** Univariate and multivariate Cox regression analyses to identify predictors for overall survival (n = 309) of PDAC patients in the original cohort

Variable	Univariate		Multivariate	
	HR (95% CI)	*P*	HR (95% CI)	*P*
Age	1.001 (0.983–1.019)	0.902		
Gender (male vs. female)	1.048 (0.729–1.509)	0.799		
BMI	1.005 (0.945–1.068)	0.884		
ASA score (1 vs. 2 vs. 3)	1.053 (0.700–1.583)	0.804		
Charlson comorbidity index (0 vs. 1 vs. ≥ 2)	0.846 (0.646–1.109)	0.226		
Year of operation (2013–2015 vs. 2016–2017)	0.708 (0.467–1.073)	0.103		
Tumor differentiation (well-moderate vs. poor)	1.909 (1.318–2.764)	0.001	2.020 (1.368–2.984)	< 0.001
Nerve invasion (yes vs. no)	1.168 (0.749–1.821)	0.494		
T stage (1 vs. 2 vs. 3)	1.459 (1.041–2.045)	0.028	1.452 (0.986–2.138)	0.059
N stage (0 vs. 1 vs. 2)	1.294 (1.009–1.659)	0.042	1.140 (0.478–2.718)	0.768
AJCC TNM stage (I vs. II vs. III)	1.334 (1.037–1.716)	0.025	1.167 (0.467–2.915)	0.740
Adjuvant treatment (yes vs. no)	0.410 (0.290–0.580)	< 0.001	0.364 (0.245–0.539)	< 0.001
Operative time	1.000 (0.999–1.002)	0.693		
Estimated blood loss	1.000 (0.999–1.001)	0.712		
Blood transfusion (yes vs. no)	1.107 (0.704–1.742)	0.659		
No. of resected lymph nodes	1.012 (0.994–1.031)	0.191		
R0 resection (yes vs. no)	0.342 (0.085–1.385)	0.133		
Surgical approach (LPD vs. OPD)	1.483 (1.031–2.135)	0.034	1.042 (0.675–1.608)	0.853

*PDAC* pancreatic ductal adenocarcinoma, *BMI* body mass index, *ASA* American Society of Anesthesiologists score, *AJCC* American Joint Committee on Cancer, *OPD* open pancreaticoduodenectomy, *LPD* laparoscopic pancreaticoduodenectomy, *HR* hazard ratio, *CI* confidence interval

## Discussion

LPD still remains technically challenging and is only performed on selected patients in a few centers. Previous reports compared the safety and efficacy between LPD and open procedures, but only a few of these earlier studies focused on the oncological outcomes of PDAC [26]. In the matched cohort of the present study, similar rates of OS were observed between the LPD and OPD groups. However, LPD was associated with shorter OS in the original cohort. This was probably due to the different baseline characteristics in the two groups, particularly the significantly lower rate of adjuvant treatment in the LPD group (39.2% vs. 70.4%), which was demonstrated as an independent risk factor for OS in the original cohort. After matching, the influences of these factors on long-term survival were almost eliminated between the two groups. To date, only one study has analyzed the oncological outcomes of these two procedures in pancreatic cancers based on propensity score [27], which involved a relatively small cohort with a 10-year surgical span, limiting the validation of its findings. In the present study, we accumulated twice as many LPD cases as the previous report within less than 5 years, and thus the statistical error as well as the impact of confounding factors, such as the improvement of overall medical care level along time, could be reduced.

Morbidity after laparoscopic surgery is an important concern and has been compared with that after open procedures by several institutions. Dokmak et al. [28] reported that LPD was associated with higher rates of severe POPF and PPH, suggesting that LPD should be performed on selected patients. In the present study, we did not find significant differences in the rates of overall POPF and grade B/C POPF between the two groups in either the original or matched cohort. Moreover, we did not observe any difference with regards to PPH. Our results are consistent with those in other studies [10, 29]. However, the POPF rate in the OPD group in our original cohort seemed to be much higher than that reported by Hackert et al. [30] (37.4% vs. 13.6%). This could be explained by the fact that an updated ISGPS definition of POPF was adopted in the present study, and our centers routinely investigated the amylase level of the drainage fluid since the first postoperative day, which could certainly increase the chance of detecting biochemical fistula. The rate of grade B/C POPF in the OPD group in our original cohort was lower than that reported by Hackert et al. [30] (5.2% vs. 12.1%). The only complication that differed between the two groups was DGE, with an obviously lower rate in the LPD group. Since the OPD and LPD cases in the present study were from two independent institutions, it is possible that this difference was caused by non-standardized postoperative management, especially with regards to the time at which nasogastric tubes were removed. Another important explanation is that no patient in the LPD group had pylorus preservation, whereas 17 (18.3%) in the OPD group in the matched cohort underwent pylorus preservation rate (*P* = 0.001). However, another study, featuring a large sample size, including 108 patients who underwent LPD and 214 patients who underwent OPD at the Mayo Clinic, also found that DGE occurred less frequently after laparoscopic surgeries, with similar pylorus preservation rates, pancreatic fistula rates, and reconstruction techniques between the two groups [15]. The mechanisms underlying this difference remain unclear. Although no significant difference was observed, the LPD group seemed to have a higher rate of wound infection. This might be ascribed to the study’s retrospective nature, and our LPD team made a detailed form to prospectively record the whole treatment course for each patient, which meant infection cases were less likely to be omitted in this group. LPD was associated with a higher rate of reoperation as compared with OPD in the original cohort (6.3% vs. 1.3%). In the OPD group, 2 patients underwent reoperations for PPH after no positive finding in DSA, and 1 for severe intra-abdominal infection with poor response to the puncture drainage and antibiotic treatments. In the LPD group, 4 patients underwent reoperations for PPH and 1 for grade C POPF. The higher reoperation rate in the LPD group might mainly result from the learning curve, which could be reflected by the fact that 4 of the 5 reoperation cases occurred in early-phase LPD cases (i.e., the first 40 cases of LPD) (reoperation rate after OPD vs. early-phase LPD, 1.3% vs. 10%, *P* = 0.008) (Table 5). In addition, it might be partly due to the differences in strategy-making between our two teams and in the ability of supportive departments between our two hospitals.

**Table 5:** Comparison of operative and postoperative characteristics of PDAC patients among OPD, early-phase LPD and late-phase LPD groups

Characteristic	OPD group (n = 230)	LPD group		*P*_*1*_ (Early- vs. late-phase LPD)	*P*_*2*_ (OPD vs. early-phase LPD)	*P*_*3*_ (OPD vs. late-phase LPD)
		Early-phase (n = 40)	Late-phase (n = 39)			
Operative time [min, median (IQR)]	260.5 (210.0–360.0)	360.0 (336.5–397.5)	260.0 (250.0–320.0)	< 0.001	< 0.001	0.284
Estimated blood loss [mL, median (IQR)]	200.0 (100.0–400.0)	200.0 (150.0–250.0)	100.0 (50.0–200.0)	< 0.001	0.180	< 0.001
Blood transfusion [cases (%)]	24 (10.4)	11 (27.5)	8 (20.5)	0.468	0.003	0.126
No. of resected lymph nodes [median (IQR)]	11.0 (7.0–15.0)	18.5 (14.3.0–26.8)	17.0 (13.0–21.0)	0.201	< 0.001	< 0.001
No. of positive lymph nodes [median (IQR)]	0 (0–2.0)	1.0 (0–2.8)	0 (0–1.0)	0.147	0.256	0.486
Positive lymph node ratio [median (IQR)]	0 (0–0.14)	0.06 (0–0.11)	0 (0–0.06)	0.110	1.000	0.154
R0 resection [cases (%)]	221 (96.1)	40 (100)	39 (100)	NA	0.426	0.438
POPF [cases (%)]	86 (37.4)	11 (27.5)	9 (23.1)	0.651	0.229	0.084
Biochemical leak	74 (32.2)	7 (17.5)	6 (15.4)	0.800	0.062	0.034
Grade B/C	12 (5.2)	4 (10.0)	3 (7.7)	1.000	0.412	0.806
DGE [cases (%)]	66 (28.7)	1 (2.5)	0 (0)	1.000^a^	< 0.001^a^	< 0.001
Grade A	27 (11.7)	0 (0.0)	0 (0)	NA	0.046	0.049
Grade B/C	39 (17.0)	1 (2.5)	0 (0)	1.000^a^	0.018	0.005
PPH [cases (%)]	14 (6.1)	4 (10.0)	2 (5.1)	0.695	0.567	1.000
Grade A	5 (2.2)	0 (0)	1 (2.6)	0.494^a^	1.000^a^	1.000^a^
Grade B/C	9 (3.9)	4 (10.0)	1 (2.6)	0.371	0.208	1.000
BL [cases (%)]	15 (6.5)	3 (7.5)	3 (7.7)	1.000	1.000	1.000
Grade A	5 (2.2)	0 (0)	0 (0)	NA	1.000^a^	1.000^a^
Grade B/C	10 (4.3)	3 (7.5)	3 (7.7)	1.000	0.646	0.619
Intra-abdominal infection [cases (%)]	21 (9.1)	2 (5.0)	2 (5.1)	1.000	0.578	0.605
Wound infection [cases (%)]	3 (1.3)	3 (7.5)	1 (2.6)	0.134	0.044^a^	0.468^a^
Other complications [cases (%)]	24 (10.4)	5 (12.5)	3 (7.7)	0.737	0.910	0.811
Overall complications [cases (%)]	151 (65.7)	25 (62.5)	14 (35.9)	0.018	0.699	< 0.001
Clavien I–II	133 (57.8)	18 (45.0)	12 (30.8)	0.193	0.132	0.002
Clavien III–V	18 (7.8)	7 (17.5)	2 (5.1)	0.169	0.098	0.792
DSA [cases (%)]	7 (3.0)	4 (10.0)	1 (2.6)	0.371	0.105	1.000
Reoperation [cases (%)]	3 (1.3)	4 (10.0)	1 (2.6)	0.371	0.008	0.468^a^
In-hospital mortality [cases (%)]	2 (0.9)	0 (0)	1 (2.6)	0.494^a^	1.000^a^	0.376^a^
90-day readmission [cases (%)]	32 (13.9)	3 (7.5)	5 (12.8)	0.681	0.265	0.855
Postoperative length of stay [days, median (IQR)]	13.0 (10.0–19.0)	13.0 (11.0–23.5)	12.0 (10.0–16.0)	0.180	0.356	0.404

*PDAC* pancreatic ductal adenocarcinoma, *OPD* open pancreaticoduodenectomy, *LPD* laparoscopic pancreaticoduodenectomy, *POPF* Postoperative pancreatic fistula, *DGE* delayed gastric emptying, *PPH* postpancreatectomy hemorrhage, *BL* bile leakage, *DSA* digital subtraction angiography, *IQR* interquartile range, *NA* not available

^a^Fisher’s exact test

As expected, a longer operative time and lower amount of estimated blood loss were evident in the LPD group, which is in line with most previous reports [31–33]. However, a controversial result was found for the high intraoperative blood transfusion rate in the LPD group. This may have mainly resulted from the aggressive management of patients in the LPD group who underwent LPD at early phase and was partly due to the different blood transfusion control regulations between our centers. Lymph node retrieval, as an important surgical parameter, has been widely compared between minimally invasive operations and open approaches. Croome et al. [11] and Asbun et al. [12] both reported that LPD was associated with a larger number of resected lymph nodes, whereas other studies reported no difference [13, 28, 29]. The present study also showed that a significantly higher number of lymph nodes were resected in the LPD group. In our experience, although this procedure is surgeon-dependent, the magnified views and flexible angles provided by laparoscopy do indeed boost aggressive lymph node dissection. Margin status was demonstrated to be an important predictor of long-term survival for PDAC patients [11, 34]. In the present study, there was a higher R0 resection rate in the LPD group with no positive margin observed, but not significantly higher than that in the OPD group. Recently, an analysis of the National Cancer Data Base performed by Torphy et al. [35] also suggested that minimally invasive pancreaticoduodenectomy was associated with a reduced odds rate for positive margins.

To date, only three randomized controlled trials (RCTs) have compared the outcomes between LPD and OPD. The first RCT, conducted by Palanivelu et al. [36], showed that LPD was associated with lower amount of blood loss, longer operative time, and a shorter length of hospital stay, and there was no significant difference between the two procedures in terms of overall complications and oncological outcomes. Similar results were reported for the PADULAP trial by Poves et al. [37] with the exception that LPD resulted in lower postoperative morbidity. The more recent LEOPARD-2 trial, which was conducted in four centers in the Netherlands, reported that the 90-day complication-related death rate was much higher in the LPD group than in the OPD group (10% vs. 2%) [38]. Thus, this trial was prematurely terminated because of safety concerns, which might be ascribed to the steep learning curve associated with this procedure. Although all participating surgeons performed at least 20 cases of LPD before the initiation of the present study, this volume seemed to be insufficient to reach the learning curve plateau. This was reflected by the fact that at least 22% of the LPD videos graded using the modified Birkmeyer scoring system were scored below the average in the LEOPARD-2 trial. The present study showed that despite the rich experience we gained in OPD, laparoscopic distal pancreatectomy, and laparoscopic central pancreatectomy, the early-phase LPD cases were associated with longer operative time, higher amount of blood loss, and, more importantly, higher overall morbidity (Table 5). This indicates that the learning curve for LPD may be much steeper than expected. A recent meta-analysis of these three RCTs concluded that LDP showed no advantage over OPD and considered that LPD could not be proposed as an equivalent alternative to OPD at present [39]. Indeed, given the complexity and safety of LPD, this procedure is inappropriate for low-volume centers. However, since the reported unfavorable results might largely attribute to that some of the surgeons might not have reached the learning curve plateau when beginning these trials, we are still confident in the implementation and promotion of LPD in high-volume pancreatic and laparoscopic centers in the future. The major issue is how to surmount the learning curve phase safely and establish an efficient and reliable training system.

There were some limitations in the present study. First, the LPD and OPD cases came from two institutions, and potential selection bias could not be avoided. However, we implemented a propensity score matching approach to counterbalance the differences in baseline characteristics between the two groups. In addition, our primary endpoint was the OS of PDAC patients, and the only two independent risk factors of OS identified through multivariable analyses were well matched between the two groups. Second, the relatively short length of follow-up limited our analyses of long-term oncological outcomes between the LPD and OPD groups. Finally, we were unable to evaluate disease-free survival in this retrospective analysis.

## Conclusions

Our analyses indicated that LPD could be technically feasible and could achieve equivalent long-term survival as compared with OPD in patients with resectable PDAC. In addition, LPD could reduce the occurrence of postoperative complications, particularly DGE. However, considering the steep learning curve and high risks involved, this procedure should be performed by experienced surgeons after adequate training in high-volume pancreatic and laparoscopic centers.

## Data Availability

The datasets used and/or analyzed during the current study are available from the corresponding author on reasonable request.

## Abbreviations

PDAC: pancreatic ductal adenocarcinoma
LPD: laparoscopic pancreaticoduodenectomy
OPD: open pancreaticoduodenectomy
RCT: randomized controlled trial
BMI: body mass index
ASA: American Society of Anesthesiologists score
OS: overall survival
DSA: digital subtraction angiography
POPF: postoperative pancreatic fistula
DGE: delayed gastric emptying
PPH: postpancreatectomy hemorrhage
ISGPS: International Study Group of Pancreatic Surgery
BL: bile leakage
CT: computed tomography
IQR: interquartile ranges
HR: hazard ratio
